# Calibration Method for Airborne Infrared Optical Systems in a Non-Thermal Equilibrium State

**DOI:** 10.3390/s23146326

**Published:** 2023-07-12

**Authors:** Mingyuan Dong, Honghai Shen, Ping Jia, Yang Sun, Chao Liang, Fan Zhang, Jinghua Hou

**Affiliations:** 1 Key Laboratory of Airborne Optical Imaging and Measurement, Chinese Academy of Sciences, Changchun 130033, China; dongmingyuan19@mails.ucas.edu.cn (M.D.);; 2 Changchun Institute of Optics, Fine Mechanics and Physics, Chinese Academy of Sciences, Changchun 130033, China; 3 University of Chinese Academy of Sciences, Beijing 100039, China; 4 Jiuquan Satellite Launch Center, Jiuquan 732750, China

**Keywords:** airborne infrared optical system, radiometric calibration, internal stray radiation, non-thermal equilibrium state

## Abstract

Airborne infrared optical systems equipped with multiple cooled infrared cameras are commonly utilized for quantitative radiometry and thermometry measurements. Radiometric calibration is crucial for ensuring the accuracy and quantitative application of remote sensing camera data. Conventional radiometric calibration methods that consider internal stray radiation are usually based on the assumption that the entire system is in thermal equilibrium. However, this assumption leads to significant errors when applying the radiometric calibration results in actual mission scenarios. To address this issue, we analyzed the changes in optical temperature within the system and developed a simplified model to account for the internal stray radiation in the non-thermal equilibrium state. Building upon this model, we proposed an enhanced radiometric calibration method, which was applied to the absolute radiometric calibration procedure of the system. The radiometric calibration experiment, conducted on the medium-wave channel of the system within a temperature test chamber, demonstrated that the proposed method can achieve a calibration accuracy exceeding 3.78% within an ambient temperature range of −30 °C to 15 °C. Additionally, the maximum temperature measurement error was found to be less than ±1.01 °C.

## 1. Introduction

Airborne infrared optical systems are commonly employed for military purposes, such as early warning, reconnaissance, and capturing infrared radiation characteristics of distant targets [1,2,3,4]. These systems utilize cooled infrared detectors in their infrared channels to detect subtle temperature variations, ensuring their suitability for high-precision radiation measurement tasks. To enable accurate measurements, performing an absolute radiometric calibration procedure is essential. This calibration procedure establishes the precise relationship between the incident radiant flux on the system and the corresponding output counts of the detectors before any measurements are taken [5]. The cooled detector incorporates the infrared focal plane array (IRFPA) within a Dewar, maintaining it at a significantly low temperature (i.e., 77 K). This cooling process ensures the detector’s high sensitivity and eliminates the impact of the external ambient temperature on the IRFPA’s responsivity and offset. In the context of airborne multi-band photoelectric infrared systems, there are two key considerations. Firstly, due to limitations in space and power on the airborne platform, these systems are designed to be extremely compact [2,3,6,7]. Secondly, the optical instrument within the IR channel exhibits high emissivity, which results in significant internal stray radiation that greatly influences the received radiation signal by the IRFPA.

When an infrared system is maintained at a constant ambient temperature for an extended duration, it will eventually achieve thermal equilibrium. At this point, the temperature of the optical instruments and their supporting structure, referred to as the “optical temperature”, will stabilize and cease to vary. However, after the airborne IR system starts to work, due to various factors such as the internal heating of the system and changes in the external environmental temperature, the system will enter a non-thermal equilibrium state. In this state, due to the influence of unsteady heat transfer, the optical temperature of each instrument inside the system will continue to change [1]. The absence of a reliable in-flight absolute radiometric calibration method precludes the observation of such changes during flight. Consequently, it becomes imperative to assess the impact of variations in internal stray radiation on the system response in the non-thermal equilibrium state through ground absolute radiometric calibration. This calibration process is of utmost importance for achieving high-precision radiometric measurements. Therefore, investigating the radiation calibration method for airborne infrared optical systems under a non-thermal equilibrium state holds practical significance.

Many studies have investigated the impact of internal stray radiation on the detector response in infrared systems. During the system design, the common approach is to construct an analysis model to assess the influence of internal stray radiation [8,9,10,11]. Analysis software such as TracePro, Lighttools, ASAP, FRED, and others are commonly used for simulation estimation. These software tools employ techniques like ray tracing and Monte Carlo methods for accurate modeling. Simulation methods are advantageous due to their ease of implementation. However, it is important to consider the deviation between the surface characteristics of the optical devices used in the calculations and the actual situation. This discrepancy can lead to significant errors in the quantitative analysis results obtained during applications. Numerous scholars have conducted studies on the detector response of uncooled detectors affected by changes in internal stray radiation. These studies primarily focus on two methods: shutter-based and shutter-less approaches. The shutter-based method utilizes the camera shutter as an approximate blackbody source and periodically captures shutter images [12]. These images are used to correct the grayscale drift. While this method allows for in-flight correction, it results in interruptions to the imaging of the target during the correction process. The shutter-less method primarily investigates the relationship between the temperatures of the key components of the uncooled camera, such as the FPA, housing, lens, etc. [13,14,15]. It employs empirical formulas to correct the responsivity and offset terms of the calibration formula during measurements. Uncooled infrared cameras are typically designed with a focus on low cost and miniaturization. The optical design complexity of the infrared channel is comparatively low. In uncooled infrared cameras, the primary source of the grayscale drift stems from changes in the ambient temperature of the camera itself. This is in stark contrast to cooled infrared cameras, rendering related methods inapplicable to those systems.

For cooled infrared imaging systems, several scholars [16,17,18,19] have analyzed the influence of internal stray radiation on the total response of the system and have established simplified models of internal stray radiation. He et al. [20] focused on analyzing the impact of key factors, such as the radiation rate, ambient temperature, and working temperature, on the temperature measurement error. In a blackbody calibration experiment, a segmented multi-parameter model of the cooled infrared thermal camera was developed using a multi-parameter calibration approach. The results showed an improvement in the temperature measurement error, particularly at lower ambient temperatures.

Hu et al. [21] developed a model to characterize the variations in the background response in a mid-infrared remote sensing camera. This model incorporates the temperatures of multiple system components during the ground calibration process. Furthermore, the authors successfully employed this model to update the background response during on-orbit operations. Chang et al. [22,23] evaluated the influence of ambient temperature on radiometric calibration in a laboratory environment. Additionally, a measurement method for assessing internal stray radiation under different ambient temperatures was proposed, enabling accurate measurement and compensation of internal stray radiation. However, in the process of establishing the aforementioned model, a simple infrared imaging system was used in the experiment. The system typically possesses a simple optical structure and readily achieves thermal equilibrium with the ambient temperature. The model was developed under the assumption of thermal equilibrium, with a single ambient temperature as the variable affecting internal stray radiation. Consequently, the model exhibits good accuracy in characterizing simple infrared systems operating under a thermal equilibrium state, but it falls short of accurately describing the internal stray radiation of non-thermal equilibrium complex systems.

To address the aforementioned issues, this article presents a radiometric calibration method specifically designed for airborne infrared radiometric systems operating under a non-thermal equilibrium state. The proposed method offers the following three advantages:

1. The established system response model takes into account the variations in internal stray radiation that occur during the unsteady heat transfer process of the system.
2. The proposed radiometric calibration method does not require the system to reach thermal equilibrium and does not introduce any additional steps compared to conventional methods.
3. The proposed method effectively reduces the error of laboratory calibration results when applied in actual measurement scenarios, particularly in cases where the optical temperature and its rate of change are high.

Section 2 introduces the radiometric calibration model for the infrared system. It discusses the analysis of the internal thermal environment of the system under different states and presents the establishment of the internal stray radiation model for a non-thermal equilibrium state. Furthermore, it proposes an enhanced radiometric calibration method and provides a calibration accuracy evaluation approach. In Section 3, the accuracy of the proposed model is validated through calibration experiments and compared with existing models. Finally, Section 4 provides the conclusions.

## 2. Inner Stray Radiation Model and Calibration Method

### 2.1. Radiometric Calibration Model

The linear response model of the cooled IRFPA serves as the foundation for infrared radiation characteristic measurement technology. In the linear region of the detector, the grayscale response can be expressed as:

(1) $$DN^{i,j}=g_{0}^{i,j}\cdot L\left(T_{b}\right)+b^{i,j}$$

where $L(T_{b})$ is the radiance of an emitter, $DN^{i,j}$ is the gray value of the (i,j)th pixel in the array, $G_{0}^{i,j}$ is the responsivity, and $B^{i,j}$ is the offset.

The model parameters are obtained through the absolute radiation calibration procedure. Commonly used calibration methods include the distant small source (DDS), distant extended source (DES), near small source (NSS), and near extended source [5]. Among these, the NES method is widely utilized due to three advantages: (1) it does not require consideration of the background, (2) it has negligible atmospheric influence, and (3) the distance between the source and the radiometer is not important. In this method, a uniform radiation source is placed at the optical window or entrance pupil of the system and is completely covered, resulting in even radiation on the IRFPA after transmission through the optical system. The standard radiation source often used in this context is a large-area blackbody radiation simulator, which exhibits characteristics such as uniformity, high emissivity, and precise temperature control. The radiance of the blackbody at temperature $T_{b}$ in the working band of the system can be determined based on the Planck formula:

(2) $$L\left(T_{b}\right)=\varepsilon L_{b}\left(T_{b}\right)=\frac{\varepsilon }{\pi }\int_{\lambda_{1}}^{\lambda_{2}}\frac{C_{1}}{\lambda^{5}}\cdot \frac{1}{e^{C_{2}/\lambda T_{b}}-1}d\lambda$$

where $L(T_{b})$ represents the radiance in the working band of the system, ε denotes the blackbody emissivity, and $C_{1}$ and $C_{2}$ are the radiation constants.

After traversing the common-aperture infrared optical system, the power of blackbody radiation received by each pixel of the detector is given by:

(3) $$\Phi_{b}^{i,j}=\tau_{opt}\frac{\pi \left(1-A^{2}\right)A_{d}D_{0}^{2}}{4f^{2}}\cdot r_{d}^{i,j}L\left(T_{b}\right)\cdot \cos^{4}\theta$$

where $\tau_{opt}$ represents the average spectral transmittance of the optical system in the corresponding wavelength band. $A_{d}$ refers to the detector area, $D_{0}$ is the pupil diameter, *f* denotes the focal length of the optical system, and $r_{d}^{i,j}$ represents the average spectral transmittance of the pixels in the corresponding wavelength band. Taking into account the presence of central obscuration in the Cassegrain system used for common aperture optics, the factor $\left(1-A^{2}\right)$ is applied, where *A* is the ratio of the diameter of the central obscuration to the diameter of the primary mirror, and θ is the angle between the chief ray of a single detector (or pixel) and the optical axis. Airborne infrared imaging systems often require a long operating distance, and as a result, they are typically designed with a small field of view (FOV) [1,22]. For a small FOV imaging system, the $\cos^{4}\theta$ term tends to approach 1. So, the IRFPA pixel’s system responsivity can be expressed as:

(4) $$g_{0}^{i,j}=\tau_{opt}\frac{\pi \left(1-A^{2}\right)A_{d}D_{0}^{2}}{4f^{2}}\cdot r_{d}^{i,j}\cdot t$$

where *t* represents the integration time.

IRFPA is composed of a large number of detection elements. Due to the limitation of the manufacturing process, each detector has a different gain $r_{d}^{i,j}$ and offset $b_{d}^{i,j}$. Usually, a two-point non-uniformity correction (NUC) program is used to achieve the consistency of the IRFPA response to uniform sources. This program makes $g_{0}^{i,j}=G$ and $b^{i,j}=B$ in Equation (1). After NUC, the linear response model at a given integration time is:

(5) $$DN=G\cdot L(T_{b})+B$$

where $L(T_{b})$ is the radiance of the standard radiation source, *G* is the system responsivity, *B* is the system offset, and DN is the gray value.

In Equation (4), $\tau_{opt}$ exhibits minimal changes within the system’s operating temperature range. $r_{d}^{i,j}$ also does not vary with the ambient temperature due to the use of a cooled detector. Therefore, it is generally assumed that *G* in Equation (5) remains largely unaffected by the ambient temperature.

Depending on the source, *B* in Equation (5) can be divided into:

(6) $$B=B_{s}+B_{0}$$

where $B_{0}$ represents the output resulting from internal factors such as the dark current and cold stop. These factors do not fluctuate with changes in the ambient temperature for a cooled detector. $B_{s}$ denotes the output caused by stray radiation.

According to the source, stray radiation can be classified into three types: external stray radiation, internal stray radiation, and narcissus. Narcissus refers to the optically reflected radiation from a cooled detector and is a form of stray radiation specific to cooled infrared systems that utilize cooled detectors. The impact of narcissus can be mitigated by reducing the reflectivity of critical surfaces [24,25], regardless of the ambient temperature. Therefore, it can be subsumed into $B_{0}$ for simplification. Additionally, the influence of external stray radiation is nearly negligible due to NES radiation calibration. None of the aforementioned factors will cause changes in the system offset *B* due to variations in the ambient temperature.

Since any object with a temperature higher than 0K emits infrared radiation, the IRFPA receives not only radiation from the target but also radiation emitted by the infrared optical system itself. Internal stray radiation has been observed to have a significant impact on the system offset, resulting in a shift in the output gray value [19,21,22]. Therefore, it is necessary to separate the internal stray radiation from the system offset and model them as separate entities.

### 2.2. Internal Stray Radiation Model

#### 2.2.1. Simplified Model of Internal Stray Radiation

The airborne infrared optical system features a compact and complex optical and mechanical structure. Every optical element along the optical path, from the main window to the infrared detector, as well as the surrounding mechanical components, serve as sources and surfaces for internal stray radiation. The IR channel optics, known for their high emissivity, contribute significantly to the thermal radiation received by the detector. The transmission of radiation undergoes refraction, reflection, and scattering by various optical and mechanical structures, rendering it highly intricate. To facilitate processing, simplified models are commonly employed for description and analysis [1,16,19].

**Figure 1:**
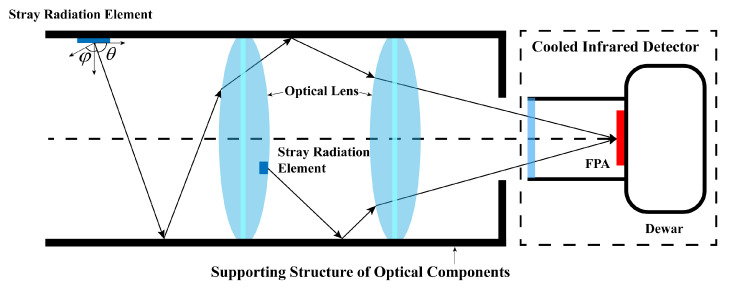
Geometry of stray radiation.

Figure 1 illustrates the intricate transmission of internal stray radiation within the system. Any radiation source that contributes to stray radiation can be subdivided into numerous micro-elements. The radiation emitted by these micro-elements undergoes multiple reflections, scattering, and refractions by various optical and mechanical surfaces before reaching the detector. The power emitted by a stray radiation element with an area dA can be expressed as follows:

(7) $$d\Phi_{s}\left(T_{s}\right)=\varepsilon \left(\theta ,\varphi \right)L\left(T_{s}\right)\cdot \Omega \cdot \tau \cdot \rho \cdot dA$$

where ε represents the in-band emissivity of the stray radiation element in a specific direction, $L(T_{s})$ represents the radiance of an ideal blackbody in the working band of the detector at the optical temperature $T_{s}$, Ω represents the projected solid angle, τ represents the total transmittance along the transmission path, and ρ represents the total reflectance along the transmission path.

The total stray radiation power received by the detector can be expressed as follows:

(8) $$\Phi_{s}=\sum_{n=1}^{N}\varepsilon_{n}\left(\theta ,\varphi \right)L\left(T_{s,n}\right)\Omega_{n}\tau_{n}\rho_{n}dA_{n}$$

where *N* represents the number of stray radiation elements. For a given system, the emissivity, transmittance, projected solid angle, and reflectivity terms in Equation (6) have been determined and vary little with the ambient temperature, making them essentially constants. Let $K_{s,n}=\varepsilon_{n}\left(\theta ,\varphi \right)\Omega_{n}\tau_{n}\rho_{n}dA_{n}$. Then, we have:

(9) $$\Phi_{s}=\sum_{n=1}^{N}K_{s,n}L\left(T_{s,n}\right)$$

Current works often assume that all stray radiating elements are at the same temperature as the ambient temperature, that is, the system is always in a thermal equilibrium state. As a result, Equation (9) can be further simplified as:

(10) $$\Phi_{s}=KL\left(T_{amb}\right)$$

where $K=\sum_{n=1}^{N}K_{s,n}$, $T_{amb}$ is the ambient temperature.

**Figure 2:**
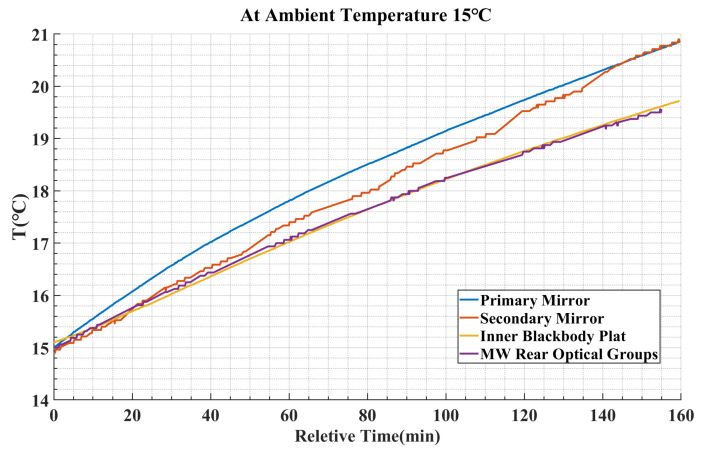
Variation curve of the optical temperature of different optical instruments at 15 °C ambient temperature.

For an infrared system with low complexity and a small number of optical devices contributing to stray radiation, it is relatively easy for the system to reach thermal equilibrium with the ambient temperature. In such cases, Equation (10) can be used to estimate the internal stray radiation of the system and meet the required level of accuracy. Figure 2 shows the changes in the optical temperature for different instruments within an airborne infrared optical system over a period of approximately 3 h, starting from the thermal equilibrium state at a 15 °C ambient temperature (system not operating) and continuing until the initiation of operation. It is evident that once the system starts functioning, factors such as internal electronic device heating and other influences disrupt the thermal equilibrium state. Moreover, the joint effect of the ambient temperature and non-thermal steady-state heat transfer within the system leads to varying gradient changes in the optical temperature. The assumption of thermal equilibrium is no longer valid. Consequently, the estimation of internal stray radiation using Equation (10) will incur progressively larger errors with increasing differences in the optical temperature among the optical instruments.

#### 2.2.2. Internal Stray Radiation Model in Non-Thermal Equilibrium State

In the case of a system in a non-thermal equilibrium state, it is theoretically possible to use Equation (9) to analyze stray radiation. However, in practical applications, it is often impractical to obtain the optical temperature of all stray radiation elements and determine the corresponding coefficients. While the assumption of thermal equilibrium cannot be applied to the entire system, it is feasible to utilize the thermal equilibrium assumption within a limited number of regions, where a reference temperature is used to characterize the stray radiation:

(11) $$\Phi_{s}=\sum_{m=1}^{M}K_{s,m}L\left(T_{s,m}\right)$$

where *M* represents the number of regions and $T_{s,m}$ is the reference optical temperature of regions. Let $G_{s,m}=K_{s,m}\cdot r_{d}\cdot t$, where $r_{d}$ is the detector’s average responsivity and *t* is the integration time. Then, we have:

(12) $$B_{s}=\sum_{m=1}^{M}G_{s,m}L\left(T_{s,m}\right)$$

Substituting Equations (6) and (12) into Equation (5), we obtain:

(13) $$DN=G\cdot L\left(T_{b}\right)+\sum_{m=1}^{M}G_{s,m}L\left(T_{s,m}\right)+B_{0}$$

Theoretically, Equation (13) can describe the response of an infrared system under a non-thermal equilibrium state.

Due to the limitations of internal space and power on airborne platforms, it is not feasible to achieve stable independent control of the optical temperature of key optical components in the infrared channel. As a result, the optical temperature changes are influenced by both the external ambient temperature and internal heating, leading to varying gradients and consistent trends, as illustrated in Figure 2. Consequently, the *M* optical temperatures in Equation (13) may exhibit a higher degree of correlation.

In multiple linear regression theory, when there is a high correlation among the explanatory variables, it can result in matrix ill-conditioning and raise concerns about the reliability of the regression results. This phenomenon is known as multicollinearity. Multicollinearity can have several implications, including inconsistent results between the analysis of variance for the entire model and the significance tests of the regression coefficients for each explanatory variable, meaningless test results for statistically significant explanatory variables, and coefficients or signs of explanatory variables that do not align with the expected physical relationships. Hence, when fitting the model and estimating the coefficients using calibration data, incorporating additional highly correlated optical temperature data will not enhance the credibility of the calibration results. Instead, it can lead to negative coefficients and reduce the model’s versatility.

In [21], the step-wise regression method was employed to select a subset of optical temperature variables, and the non-negative least squares method was utilized to ensure that the obtained coefficients were non-negative and aligned with the physical meaning. However, it is worth noting that step-wise regression adds or removes variables in a specific order, which can lead to the exclusion of important variables in the analysis. Additionally, this method requires a sufficiently large sample size to ensure the reliability of the regression results. To address these challenges, this article proposes the use of variance inflation factor (VIF) analysis in conjunction with systematic practices for selecting the variable subset in the model. By incorporating systematic practices and considering the specific needs of radiometric calibration procedures, the proposed method aims to achieve a robust selection of optical temperature variables without requiring a large number of samples. VIF can be expressed as:

(14) $$VIF_{i}=\frac{1}{1-R_{i}^{2}},i=1,2,3,\cdots ,p$$

where $VIF_{i}$ is the variance inflation factor of the i-th explanatory variable, *p* is the number of explanatory variables in the multiple regression model, and $R_{i}$ is the R2 statistic from the regression of the i-th independent variable on the other covariates. If there is a high correlation between the explanatory variables with high VIF, then:

(15) $$a_{i}X_{i}=\sum_{j\neq i}a_{j}X_{j}+\varepsilon$$

where X represents the explanatory variable (optical temperature), *a* represents the coefficient, and ε represents the random error. It is important to note that in this context, we are assuming the presence of a relatively significant linear relationship among the explanatory variables (as shown in Figure 2). By employing Equation (15), it becomes possible to screen out explanatory variables with low correlations. Furthermore, this equation aids in the selection of one high-correlation explanatory variable that can be added to the subset of variables, addressing the issue of multicollinearity.

For ease of description, we simplify the characterization of the internal thermal environment of a system into three distinct states:

State (a): The system is not operational and remains in thermal equilibrium. During this state, the entire system shares the same temperature as the ambient temperature.

State (b): When the system is powered on, it transitions into a non-thermal equilibrium state. Under the influence of various factors, the system undergoes continuous changes and experiences unsteady heat transfer both internally and externally.

State (c): The system eventually reaches a new thermal equilibrium state during operation.In this state, the optical temperature within the system may differ from the ambient temperature, and an uneven distribution of temperatures can exist. However, these temperature distributions remain constant and do not undergo further changes.

Based on actual flight data, it is hard for the system to reach State (c) within a mission cycle. Typically, the optical temperature continuously varies during the measurement process.

When the system is in State (a), the expression for stray radiation is provided by Equation (10). In this state, the optical temperature of the system is equivalent to the ambient temperature. Therefore, the coefficient term in the response model solely pertains to the structural and surface characteristics of the optical system. When the system transitions to State (b), it becomes necessary to screen a subset of variables in order to obtain a reliable fitting result. Equation (15) introduces the coefficient *a* when expressing high VIF explanatory variables. Notably, the coefficient *a* is associated with all explanatory variables. This implies that the coefficient of the internal stray radiation term in State (b) is not solely influenced by the structure and surface characteristics of the optical system as it is in State (a). Instead, it also depends on the reference optical temperature in different regions. Consequently, the same coefficient used in State (a) cannot be applied in State (b). To differentiate between the internal stray radiation in State (a) and State (b), we express them using absolute and relative representations, respectively. As a result, we propose a novel radiation calibration model as follows:

(16) $$DN=G\cdot L\left(T_{b}\right)+\sum_{q=1}^{Q}\left[G_{s1,q}\cdot L\left(T_{0,q}\right)+G_{s2,q}\cdot \Delta L_{q}\right]+B_{0}$$

where $T_{0,q}$ is the optical temperature in thermal equilibrium, $\Delta L_{q}=L\left(T_{s,q}\right)-L\left(T_{0,q}\right)$, $T_{s,q}$ is the optical temperature at the time of measurement, and *Q* is the number of remaining explanatory variables after variable filtering.

### 2.3. Calibration Method and Accuracy Evaluation

Based on the previous analysis, we propose Equation (16) as the radiation calibration formula for the system in a non-thermal equilibrium state. In airborne infrared optical systems, multiple optical filters and neutral-density filters are commonly used in the IR channel. These filters serve to expand the dynamic range of the system and acquire more comprehensive information on the radiation characteristics. By employing such filters, the system can effectively enhance its performance and capture a wider range of radiation data.

The combination of these filters and camera integration time can reach more than 100 conditions, so performing a radiometric calibration experiment for all conditions of the infrared channel at one ambient temperature usually takes several hours. Similar to the actual measurement task, the optical temperature inside the system will continue to change within hours of performing the absolute radiation calibration process. Combined with the radiation calibration model established in this article, we propose a calibration method to determine the coefficient of absolute radiation:

1. Perform the NES radiation calibration procedure by utilizing a blackbody as the standard radiation source. This calibration should be conducted at a minimum of two ambient temperatures. It is essential to ensure that the gray value falls within the linear range of the detector when capturing blackbody images. Additionally, multiple temperature measurement sensors are set in the system, and the optical temperature information is recorded concurrently with image collection.
2. Conduct VIF analysis on the collected optical temperature information. Combine this analysis with the actual system setup to screen the optical temperature variables and determine the final radiation calibration model to be employed.
3. Fit the data to obtain the coefficients of absolute calibration.

Radiometry and thermometry are the reverse processes of radiometric calibration. Once the grayscale value of the target is obtained, it can be incorporated into Equation (16) to calculate the target’s radiance or intensity. The disparity between the target radiance and the actual value is defined as the calibration error, which serves as a metric for evaluating the calibration accuracy. Thus, the calibration error is calculated as follows:

(17) $$E_{c}=\frac{\hat{L}\left(T\right)-L\left(T\right)}{L(T)}\times 100\%$$

where $\hat{L}\left(T\right)$ represents the blackbody radiance calculated according to Equation (16) and L(T) is the theoretical blackbody radiance. To assess the accuracy of the calibration formula across various ambient temperatures, the radiation calibration accuracy of the model at a specific ambient temperature is defined as follows:

(18) $$E_{e}=\max\left(\left|E_{c}\left[N\right]\right|\right)$$

where $E_{c}\left[N\right]$ represents the *N* calibration errors calculated using Equation (17) for the verification data. The acceptable system absolute radiation calibration error should be within 10% [22].

## 3. Analysis of the Calibration Method

### 3.1. Experiments

**Figure 3:**
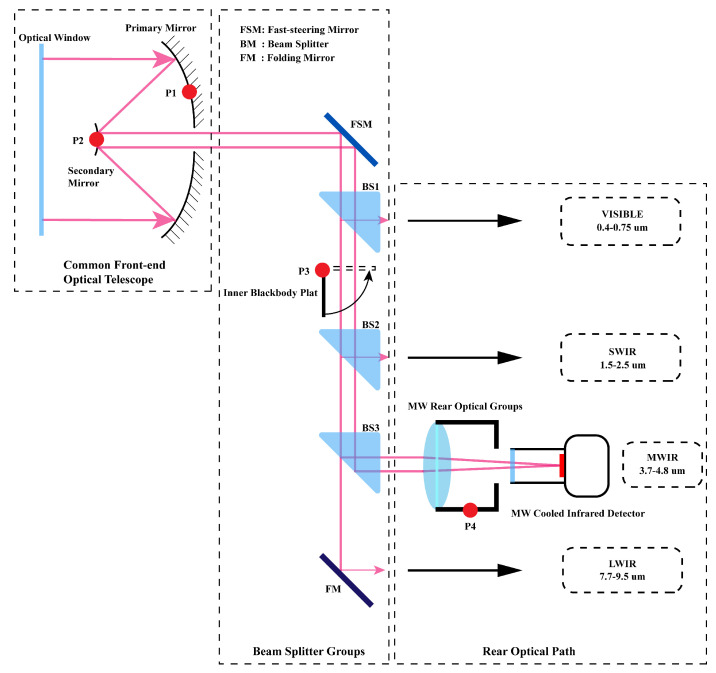
Illustration of optical configuration. Tmp117 temperature sensors are set at the P1–P4 positions in the system to obtain the optical structure temperature.

To validate the efficacy of the model, several radiation calibration experiments were conducted at various ambient temperatures. The airborne infrared optical system employed in these experiments consists of a Cassegrain objective lens, multiple beam splitters, and four channels for VIS (0.4–0.75 μm), SWIR (1.5–2.5 μm), MWIR (3.7–4.8 μm), and LWIR (7.7–9.5 μm). Figure 3 provides an illustration of the optical structure of the system. Within the MWIR channel, there is a cooled mid-wave infrared detector, manufactured by the Shanghai Institute of Technical Physics of the Chinese Academy of Sciences (SITP). The detector features a resolution of 640 × 512 and a pixel size of 15 um. The camera’s NETD@25 °C is typically lower than 22 mK, and its response band ranges from 3.7 to 4.8 μm. The camera produces a 14-bit grayscale image, while its field of view (FOV) measures 3° × 3°.

In Figure 3, the TMP117 temperature sensor is positioned at locations P1–P4 to measure the optical temperature of the medium-wave infrared channel optical device. This sensor offers a temperature measurement accuracy of ±0.15 °C within a temperature range of −40 °C to 70 °C.

To fulfill the requirements of the ambient temperature experiment and NES radiation calibration, we utilized CI systems’ SR-800N-20D-CH-ET blackbody. The blackbody possesses an emissivity of 0.98 ± 0.02 and a surface source size of 500 × 500 mm, which sufficiently covers the system aperture. It can be utilized within the temperature test chamber, offering a working temperature range of −40 to 80 °C. The blackbody itself operates within a temperature range of −15 °C to 100 °C, with a temperature control accuracy of better than 0.015 °C and temperature stability of better than ±0.008 °C.

To mitigate the potential introduction of errors resulting from intervening optical windows, we implemented a scheme where both the blackbody and the entire airborne infrared optical system were placed within a 60 $m^{3}$ temperature test chamber. The temperature test chamber had a temperature control range of −60 to 90 °C, with a temperature control accuracy of better than 1 °C. However, achieving uniformity in the internal temperature field was challenging due to the chamber’s large volume. To address this issue, a temperature sensor was installed near the system to obtain accurate ambient temperature readings.

**Figure 4:**
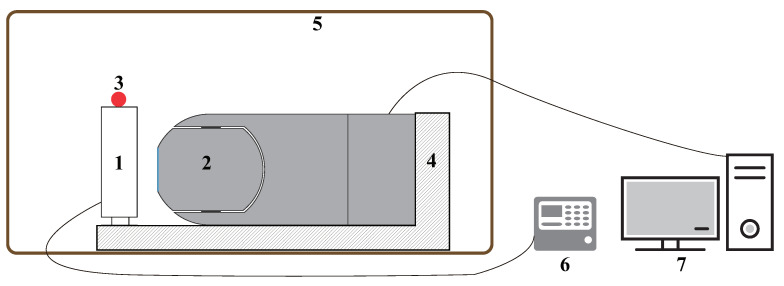
Experimental setup for radiometric calibration: 1—blackbody; 2—airborne infrared optical system; 3—ambient temperature sensor; 4—support plate; 5—temperature test chamber; 6—blackbody controller; 7—data processing system.

Figure 4 depicts the equipment and facilities utilized in the calibration experiment. The procedure involved placing the entire system and the blackbody inside an environmental incubator. The blackbody was positioned in close proximity to the system window, enabling efficient heat transfer. Calibration data were collected at four specific ambient temperatures: −25 °C, −5 °C, 5 °C, and 15 °C. The specific steps for the calibration experiment are outlined below:

Step 1: Once the chamber reaches the desired set ambient temperature, allow the system to cool for a minimum of 3 h without any operation. This ensures that the internal optical temperature of the system aligns with the ambient temperature, thereby reaching State (a).

Step 2: Initiate the system and transition it into State (b). Set the blackbody temperature to 20 °C. Collect separate images for all the working conditions by combining the seven integration time gears of the MW, four filter gears, and two neutral-density filter gears. It is important to adhere to the manufacturer’s recommendations and ensure that the average gray value of the acquired image falls within the interval of [3800, 13,200]. This range guarantees the linearity of the detector’s response.

Step 3: Repeat the process outlined in Step 2 for every 10 °C increment within the range of 20 °C to 70 °C until all calibration data are collected. Once all the data for a specific temperature point have been gathered, adjust the environmental thermostat to the next temperature point and repeat Step 1 to Step 3 at that particular temperature. Continue this process until data collection is complete for all desired temperature points.

It is worth noting that the data processing system recorded the temperature information of the temperature measurement point at the time of image acquisition in the specially designed image auxiliary information. Furthermore, all collected images underwent one-point NUC to mitigate spatial non-uniformity. To address temporal non-uniformity, dozens of frames of images were captured at a frame rate of 25 Hz during each data collection. The mean gray value of the multi-frame images was used as the system response corresponding to the blackbody temperature. Following the aforementioned steps, the calibration dataset was obtained. Subsequently, data for the test dataset were collected in the same manner, acquiring data at ambient temperatures of −30 °C, −25 °C, −10 °C, −5 °C, 5 °C, 10 °C, and 15 °C.

### 3.2. Selecting Optical Temperature Variables Subset

To analyze the multicollinearity of the optical temperature variables, the calibration data collected at ambient temperatures of −25 °C, −5 °C, 5 °C, and 15 °C were utilized. The dataset comprised the following variables: the blackbody temperature (X0) at the time of image collection, as well as temperature data from four measurement points: X1 (primary mirror), X2 (secondary mirror), X3 (inner blackbody plate), and X4 (MW rear optical groups). The VIF was calculated for these variables using Equation (14).

Table 1 presents the VIF values calculated for the different images collected at −25 °C, −5 °C, 5 °C, and 15 °C ambient temperatures using the blackbody temperature as an independent variable. It is generally accepted that if the VIF value exceeds 100, the explanatory variable exhibits severe multicollinearity with other variables [26]. Upon examining the calculation results, it was evident that there existed a strong linear relationship among the X1–X4 variables. In contrast, the blackbody temperature (X0) demonstrated no relationship with the ambient temperature or the internal temperature of the system. Its VIF value was considerably low, aligning with the actual scenario.

**Figure 5:**
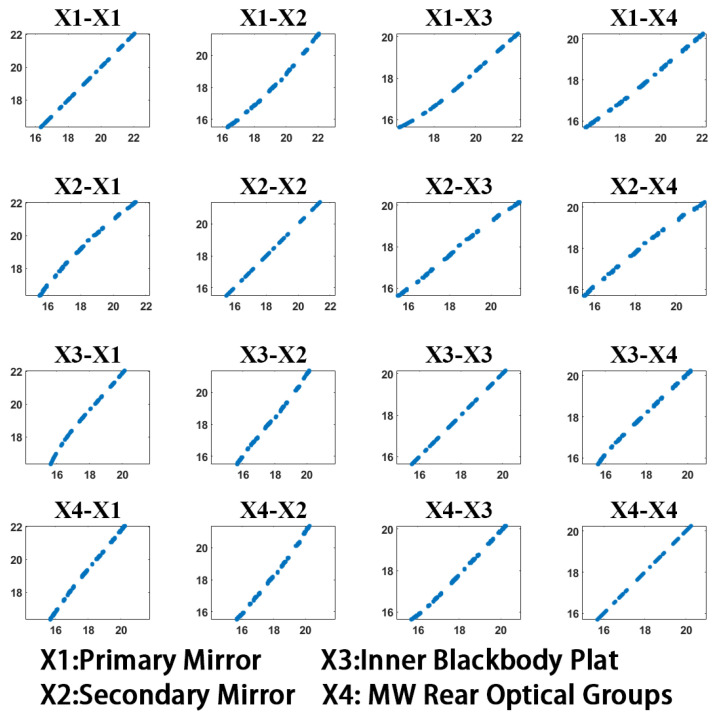
Scatter diagrams between X1 and X4.

Taking the data at 15 °C as an example, we created scatter diagrams between X1 and X4, as shown in Figure 5. The figure clearly illustrates a distinct linear relationship between the two different optical temperature variables. This observation confirms that our assumption, made when employing Equation (15), is valid.

In this article, the airborne infrared optical system employed a common aperture through which the incoming light was divided by several beam splitters. Subsequently, the separated light passed through individual optical paths for each band and entered the corresponding detector. Therefore, the temperature sensor set in P4 was deemed to be a more representative indicator of internal stray radiation in the radiation calibration model. Notably, the VIF value associated with this optical temperature variable was the lowest among all the variables considered. Based on these findings, the final radiation calibration model was established by utilizing the optical temperature measured at P4 as the sole reference temperature. The model can be expressed as:

(19) $$DN=G\cdot L\left(T_{b}\right)+G_{s1}\cdot L\left(T_{0}\right)+G_{s2}\cdot \Delta L+B_{0}$$

where $T_{0}$ is the measured value of the P4 temperature sensor in State (a), and $\Delta L=L\left(T_{s}\right)-L\left(T_{0}\right)$, $T_{s}$ represents the measured value of the P4 temperature sensor at the moment of image collection.

### 3.3. Accuracy Analysis

To verify the validity of Equation (19), which corresponds to the radiation calibration model, a comparison of two models was performed, as shown in Table 2:

Model 1 used the ambient temperature $T_{amb}$ to represent the optical temperature of the system, without considering the change in internal stray radiation. At the beginning of each experiment, the system reached thermal equilibrium after sufficient heat exchange with the surrounding environment without working, so $T_{amb}=T_{0}$. Model 2 used $T_{s}$ to represent the measurement value of the P4 temperature sensor at the time of image collection, considering the change in internal stray radiation. However, it assumed that the internal optical temperature of the system at the measurement time was uniform and equal to $T_{s}$. In other words, the system was considered to be approximately in thermal equilibrium at each measurement time.

**Figure 6:**
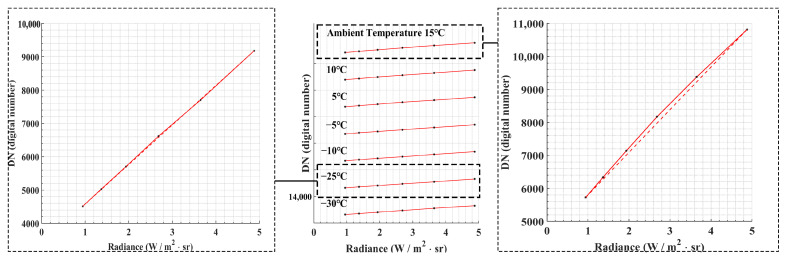
Original data collected at 7 ambient temperatures. The system works in the 3.7–4.8 μm band using a neutral-density filter with a transmittance of 99%, and the integration time of the MW infrared camera is 6 ms.

To illustrate this point, consider the data obtained from the 3.7–4.8 μm wave band using a neutral-density filter with 99% transmittance and an integration time of 6 ms. Figure 6 depicts the original data collected following the experimental steps outlined in Section 3.1. It is evident that the original data, recorded at an ambient temperature of 15 °C, exhibited pronounced nonlinearity. However, at an ambient temperature of −25 °C, the nonlinearity in the response was significantly reduced, indicating that the detector’s performance was not the cause of this nonlinearity. Instead, it can be attributed to internal stray radiation variations that occurred during the measurement process. The extent of nonlinearity became more apparent as the contributions from internal stray radiations increased and the gradients of change became larger.

To investigate the impact of internal stray radiation changes at different ambient temperatures, we divided the analysis based on an ambient temperature of 0 °C. The calibration formula was then fitted separately using the data from two ambient temperatures. The fitting coefficients of the three models are shown in Table 3.

**Figure 7:**
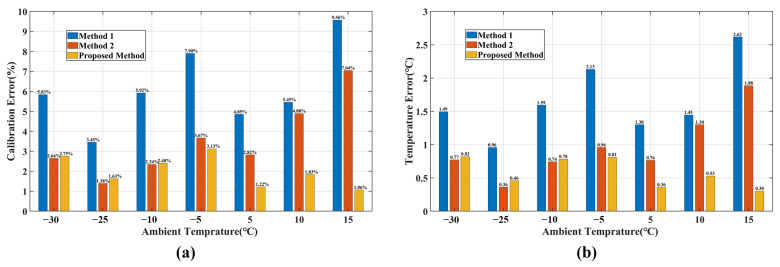
(**a**) Calibration error and (**b**) temperature measurement error. The system works in the 3.7–4.8 μm band using a neutral-density filter with a transmittance of 99%, and the integration time of the MW infrared camera is 6 ms.

Based on Equations (17) and (18), the calibration accuracy and temperature measurement error of the system under seven ambient temperatures were calculated, as shown in Figure 7. According to the findings shown in Figure 7, the proposed method achieved a calibration accuracy of better than 3.13% and a maximum temperature measurement error of less than 0.82 °C across the seven ambient temperatures. In comparison, Model 1 exhibited a calibration accuracy of 9.56% and a maximum temperature measurement error of 2.62 °C, whereas Model 2 demonstrated a calibration accuracy of 7.04% and a maximum temperature measurement error of 1.88 °C. This indicates that the proposed method surpassed both Model 1 and Model 2 in terms of calibration accuracy and temperature measurement precision.

**Figure 8:**
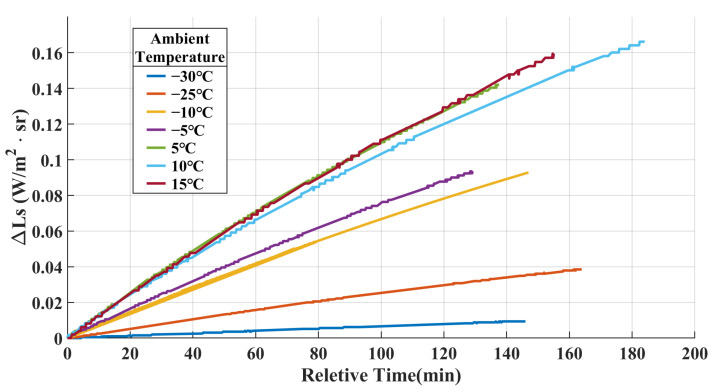
MW rear optical group temperature curves at different environmental temperatures during the execution of calibration experiments.

Upon further analysis, it was observed that when the ambient temperature was below 0 °C, the proposed method and Model 2 exhibited similar accuracy. However, as the ambient temperature increased above 0 °C, the error of Model 2 increased with the rising ambient temperature, whereas the error of the proposed method remained stable and maintained better accuracy compared to Model 2. This difference can be attributed to the variations in the intensity and gradient of stray radiation within the system under different ambient temperatures, as illustrated in Figure 8.

When the ambient temperature was relatively low, the contribution of internal stray radiation was minimal, and the gradient of change was low. As discussed in Section 2.2.2, the system was considered to be in a thermal equilibrium state during the measurement time. This equilibrium state was represented by the small difference between the coefficients $G_{s1}$ and $G_{s2}$ in the calibration formula of the proposed method, as shown in Table 3. The following relationship can be obtained:

(20) $$G_{s1}\cdot L\left(T_{0}\right)+G_{s2}\cdot \Delta L\approx G_{s}^{\prime }\left(L\left(T_{0}\right)+\Delta L\right)=G_{s}^{\prime }L\left(T_{s}\right)$$

Therefore, when $T_{amb}<0$, both the proposed model and Model 2 exhibited similar accuracies. However, when $T_{amb}>0$, the intensity and gradient of internal stray radiation increased significantly. Applying the thermal equilibrium approximation to address the internal stray radiation in a non-thermal equilibrium state would introduce a significant error. This discrepancy was reflected in the noticeable difference between the coefficients $G_{s1}$ and $G_{s2}$ in the calibration formula of the proposed method, as shown in Table 3. In contrast, Model 2 lacked the capability to describe the changes in stray radiation within the system under a non-thermal equilibrium state, leading to a significant increase in the error. Conversely, the proposed model maintained a high level of accuracy. The experimental results clearly demonstrate the superiority of the proposed model in handling the issue of internal stray radiation in non-thermal equilibrium systems.

**Figure 9:**
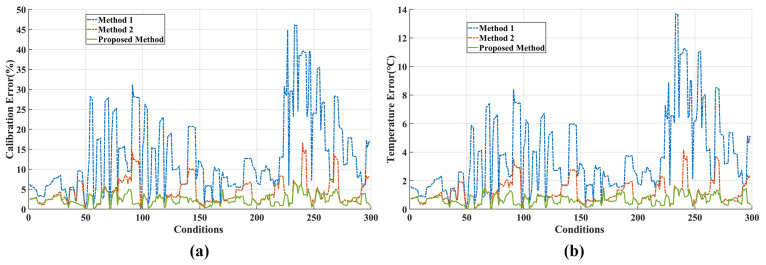
(**a**) Calibration errors and (**b**) temperature measurement errors for all conditions.

To assess the overall calibration accuracy of the proposed model, we considered the combination of ambient temperature, optical filter, neutral-density filter, and integration time as conditions. By calculating the radiometric calibration accuracy and maximum temperature error for all conditions, we could compare the performance of the three models. The results are presented in Figure 9. By grouping the optical filters, we could compile the statistics of the maximum calibration errors and temperature measurement errors for the three calibration models within the ambient temperature range of −30 °C to 15 °C. The results are summarized in Table 4.

Based on the findings in Figure 9 and Table 4, it can be seen that the radiation calibration model obtained through the proposed method achieved a radiation calibration error of better than 6.83% within the ambient temperature range of −30 °C to 15 °C. Additionally, the temperature measurement error was better than ±1.56 °C. In contrast, Model 1, which did not consider the changes in internal stray radiation, exhibited errors reaching 46.12% for certain conditions. Similarly, Model 2, which neglected the influence of unsteady heat transfer, exhibited errors as high as 16.57% for certain conditions. In the commonly used 3.7–4.8 μm band and within the ambient temperature range of −30 °C to 15 °C, the radiation calibration accuracy of the proposed model exceeded 3.87%, whereas the temperature inversion error was better than ±1.01 °C. In comparison, Model 1 achieved a calibration accuracy of 13.12% for the same conditions, whereas Model 2 achieved an accuracy of 8.32%. Furthermore, the maximum temperature measurement errors for Model 1 and Model 2 were ±3.64 °C and ±2.28 °C, respectively.

The experimental results highlight the high radiometric calibration accuracy achieved by the proposed method for airborne infrared radiometry systems. The derived radiation calibration formula exhibited stable and accurate performance within the designated working temperature range of −30 °C to 15 °C. Notably, the proposed method excelled in characterizing the internal stray radiation present in complex infrared systems operating in non-thermal equilibrium states. Particularly, when dealing with scenarios featuring high optical temperatures and significant gradients of change, the proposed method outperformed existing methods with remarkable accuracy improvements.

## 4. Conclusions

In this article, a calibration method suitable for airborne infrared optical systems in a non-thermal equilibrium state is proposed based on extensive research on internal stray radiation. The analysis of the optical temperature change in the system leads to the development of a reference optical temperature selection strategy. Additionally, a calibration model is provided to accurately describe the variations in internal stray radiation within the non-thermal equilibrium system. The experimental results validate the effectiveness of the proposed method in capturing the internal stray radiation variations under different ambient temperatures, without the need for additional experimental steps. This enhances the practical applicability of laboratory calibration results. The proposed method demonstrates a significant improvement in calibration accuracy compared to existing methods. While the effectiveness of the method was validated using MW channel data for a specific system, it is important to note that this method can also be applied to other MWIR or LWIR systems.

## Figures and Tables

**Table 1:** VIF results for the optical temperature variables.

Ambient Temperature (°C)	VIF X0	VIF X1	VIF X2	VIF X3	VIF X4
−25	5.40	710.32	870.32	483.76	282.10
−5	4.22	498.46	985.94	1036.65	208.61
5	3.54	5100.02	547.60	6060.65	199.28
15	3.47	449.40	442.41	1116.84	309.57

**Table 2:** Model comparison.

Model	Formula
Model 1	$DN=G\cdot L\left(T_{b}\right)+G_{s}L\left(T_{amb}\right)+B$
Model 2	$DN=G\cdot L\left(T_{b}\right)+G_{s}L\left(T_{s}\right)+B$
Proposed model	$DN=G\cdot L\left(T_{b}\right)+G_{s1}\cdot L\left(T_{0}\right)+G_{s2}\cdot \Delta L+B$

**Table 3:** The fitting coefficients of the three models.

**Model 1**	*G*	$G_{s}$	*B*	/	$T_{amb}$ range
	1167.36	2183.24	3082.49	/	$T_{amb}<0$
	1239.55	2286.04	2803.12	/	$T_{amb}\geq 0$
**Model 2**	*G*	$G_{s}$	*B*	/	$T_{amb}$ range
	1136.83	2416.11	3013.31	/	$T_{amb}<0$
	1176.61	1856.76	3132.85	/	$T_{amb}\geq 0$
**Proposed** **model**	*G*	$G_{s1}$	$G_{s2}$	*B*	$T_{amb}$ range
	1133.39	2381.02	2688.03	3022.17	$T_{amb}<0$
	1049.10	1735.06	5618.23	3275.59	$T_{amb}\geq 0$

**Table 4:** Statistics of calibration errors and temperature measurement errors under different optical filters.

Wave Band (μm)	Calibration Error (%)			Maximum Temperature Error (°C)		
	Proposed Model	Model 1	Model 2	Proposed Model	Model 1	Model 2
3.7–4.8	3.87	13.12	8.32	1.01	3.64	2.28
3.6–4.1	6.83	46.12	16.57	1.56	13.70	4.13
4.3–4.5	5.08	39.66	13.29	1.40	11.06	3.69
4.5–4.8	4.38	20.99	8.24	1.46	5.40	2.37

## Data Availability

The data presented in this study are available upon request from the corresponding author.
